# Mechanism of Si Ni Decoction in Stabilizing Atherosclerotic Plaques in the Lower Extremities by Regulating Macrophage Polarization via NCOR1 Mitophagy

**DOI:** 10.1002/iid3.70546

**Published:** 2026-10-25

**Authors:** Zhe Liu, Shuhui Ge, Jianfei Yang, Ying Zhou, Li Jin, Yudong Fang

**Affiliations:** ^1^ Department of Vascular Diseases, Shanghai TCM‐Integrated Hospital Shanghai University of Traditional Chinese Medicine Shanghai China; ^2^ School of Pharmacy Macau University of Science and Technology. Avenida Wai Long Taipa China

**Keywords:** atherosclerotic plaques, CV, macrophage polarization, mitophagy, SND

## Abstract

**Introduction:**

Si Ni decoction (SND) is known for its anti‐inflammatory, immune‐regulating, and microcirculation‐improving effects. However, the molecular mechanisms underlying its role in atherosclerosis (AS) remain unclear. It aimed to explore the mechanisms by which SND stabilizes lower extremity atherosclerotic plaques through the regulation of macrophage polarization via mitophagy.

**Materials and Methods:**

AS models were established in RAW 264.7 macrophages stimulated by lipopolysaccharide (LPS) and assigned into five groups [blank (BG), model (MG), low‐dose (LDG), medium‐dose (MDG), and high‐dose (HDG)]. The expression of pro‐inflammatory and anti‐inflammatory cytokines, M1 and M2 macrophage markers, mitophagy‐related proteins, and cell viability (CV) were detected in each group.

**Result:**

Relative to the MG, in the LDG, MDG, and HDG, with increasing drug dosage, the levels of pro‐inflammatory cytokines interleukin‐6 (IL‐6) and tumor necrosis factor‐α (TNF‐α) decreased gradually, while the degree of IL‐10 raised; CD16/32 and COX2 were visibly reduced, and Arg1, CD206 visibly elevated; LC3, Parkin, FUNDC1 were visibly reduced, and P62 and CV were obviously elevated (*p* < 0.05). Meanwhile, SND dose‐dependently reversed the downregulation of NCOR1 expression induced by LPS (all *p* < 0.05).

**Conclusion:**

The results of the present in vitro cell experiments showed that SND upregulated NCOR expression and improved abnormal mitophagy, thereby regulating macrophage polarization, alleviating inflammatory damage, and restoring mitochondrial function, thus providing preliminary in vitro experimental evidence for intervening in inflammatory lesions associated with atherosclerosis.

**Aim:**

The present study aimed to investigate the in vitro mechanism by which SND regulates macrophage polarization through the mitophagy pathway, thereby elucidating its intervention effect on atherosclerosis‐related inflammatory damage. The specific objectives are as follows: (1) To reveal the potential mechanisms of SNT in regulating inflammatory responses by detecting the expression levels of pro‐inflammatory cytokines (such as IL‐6, TNF‐α) and anti‐inflammatory cytokines (such as IL‐10) in cells. (2) To further elucidate the regulatory effects of SNT on macrophage polarization by comparing the expression levels of M1‐type (such as CD16/32, COX2) and M2‐type (such as Arg1, CD206) macrophage markers after intervention with different doses of SNT. (3) To uncover the regulatory effects of SNT on macrophage function through the mitochondrial autophagy pathway by detecting the expression of mitochondrial autophagy markers (such as LC3, Parkin, P62, and FUNDC1) via Western blotting. (4) To assess the potential of SNT in improving mitochondrial function by measuring the OCR and ECAR of cells using the Seahorse XF real‐time cell analyzer. (5) To further evaluate the role of SNT in maintaining cell viability by detecting its effects on cell proliferation and survival rate using the MTS assay.

**Core Tips:**

Atherosclerosis models were established in RAW 264.7 macrophages stimulated by LPS, and interventions with different doses of SND were applied. The experimental groups included the blank group, model group, and different doses of SND groups. Real‐time PCR was used to detect the gene expression of pro‐inflammatory cytokines (IL‐6, TNF‐α) and anti‐inflammatory cytokines (IL‐10); Western blotting was used to detect the expression of M1 and M2 macrophage markers and mitochondrial autophagy‐related proteins; the Seahorse analyzer was used to detect mitochondrial function; and the MTS method was used to assess cell viability. The present study suggests that SND attenuates inflammatory damage, restores mitochondrial function, and modulates macrophage polarization via regulation of NCOR1 and aberrant mitophagy at the in vitro cellular level. This study provides new molecular mechanisms for the potential application of SND in atherosclerosis and offers experimental evidence for clinical treatment.

## 1 Introduction

Lower extremity atherosclerosis (LEA), a major subtype of peripheral artery disease (PAD), manifests as progressive arterial stenosis, vascular sclerosis, and thrombotic occlusion in the lower extremity vasculature, leading to insufficient blood flow and various clinical symptoms such as intermittent claudication, muscle weakness, pain, and severe ischemic ulcers and necrosis [1, 2, 3]. LEA not only visibly affects patients' quality of life but also serves as a critical risk amplifier for systemic cardiovascular morbidity, making it a serious public health issue [4]. With the advent of an aging society and the increasing prevalence of hyperlipidemia, etc., the incidence of LEA is rising and has become one of the important diseases affecting global health. The pathological features of LEA mainly include lipid deposition in the arterial intima, proliferation of smooth muscle cells, fibrous tissue formation, and infiltration of immune cells such as macrophages, ultimately leading to plaque formation. These plaques can cause stenosis of the vascular lumen, and in severe cases, thrombosis, leading to tissue ischemia or necrosis [5]. Plaque rupture and thrombus formation are the main determinants of cardiovascular events, with macrophages playing a crucial role in this process. Macrophages play different roles at different stages of AS, especially in plaque formation, stabilization, and rupture, where their polarization state visibly affects plaque stability [6].

In terms of treatment, although pharmacological therapies (such as statins and antiplatelet drugs) have been widely used in the clinical management of LEA, many challenges remain [7, 8, 9]. For example, existing treatment regimens can slow disease progression to some extent but often fail to completely reverse plaque formation and stabilization, and may be accompanied by certain side effects. Therefore, the search for new therapeutic targets and strategies, especially those that stabilize plaques by regulating immune and inflammatory responses, has become one of the hotspots in recent research [10]. SND originates from the *Treatise on Febrile Diseases* and is known for its warming, blood‐activating, and circulation‐improving effects. It has been used historically to treat cold syndromes caused by yang deficiency, such as abdominal pain due to cold‐dampness [11, 12]. In recent years, with the modernization of traditional Chinese medicine, SND has been widely used as an adjunct therapy for various cardiovascular diseases. Extensive studies have shown that SND not only regulates cardiovascular function but also improves blood circulation, inhibits inflammatory responses, and enhances immunity. Its action in AS positions it as a novel candidate for precision medicine strategies in vascular pathologies [13]. Research has found that SND can slow the progression of AS through multiple pathways, including regulating endothelial cell function, inhibiting macrophage activation, and reducing blood lipids [14]. However, the specific mechanisms by which SND regulates macrophage polarization and plaque stability are still not fully understood and require further investigation.

An increasing body of evidence has demonstrated that nuclear receptor corepressor 1 (NCOR1), as a key transcriptional corepressor, plays a critical role in both macrophage inflammatory phenotype switching and mitochondrial homeostasis regulation. Oppi et al. [15] found that macrophage‐specific NCOR1 deficiency significantly exacerbated atherosclerotic lesions in mice, as manifested by increased foam cell formation, upregulated pro‐inflammatory cytokine expression, enlarged necrotic core, and thinner fibrous cap. Mechanistically, NCOR1 exerts its protective effects mainly by inhibiting the pro‐atherogenic activity of peroxisome proliferator‐activated receptor gamma (PPARγ). Notably, NCOR1 expression levels are decreased in human atherosclerotic plaques, and the reduction is more pronounced in ruptured plaques than in non‐ruptured ones. In a complementary study, Chen et al. [16] provided additional evidence that NCOR1 is involved in regulating glucose metabolic reprogramming in macrophages, a process closely associated with the development and progression of atherosclerosis. Despite the above evidence highlighting the pivotal role of NCOR1 in atherosclerosis, it remains unclear whether the anti‐inflammatory and functional modulatory effects of Sini Decoction (SND) on macrophages are also mediated through NCOR1. To address this research gap, the present study identifies NCOR1 as a potential pathway target and attempts to explore the molecular mechanism of SND. Specifically, we aim to observe the regulation of NCOR1‐related mitophagy and macrophage polarization by SND at the in vitro level, thereby elucidating the possible pathways through which SND alleviates inflammatory injury in atherosclerosis.

## 2 Materials and Methods

### 2.1 Experimental Cells

RAW 264.7 macrophage cell line was selected as the research subject. The cells were provided by Shanghai TCM‐Integrated Hospital and were authenticated to meet the experimental requirements. The study strictly followed the laboratory biosafety regulations to ensure that the cell culture and handling processes complied with relevant experimental standards and ethical principles.

### 2.2 As Model in Cells

AS models were established in RAW 264.7 cells by stimulating with LPS. RAW 264.7 cells were carefully seeded into the plate with 6 wells, cultured in a high‐glucose DMEM medium, which was supplemented with 10% fetal bovine serum and 1% penicillin/streptomycin. They were maintained at a constant temperature of 37°C in an incubator with a humidified atmosphere of 5% CO_2_, ensuring optimal growth conditions and CV. When they reached approximately 70%‐80% confluence, they were washed 1–2 times with serum‐free medium, followed by stimulation with 1 μg/mL LPS for 24 h.

### 2.3 Experimental Grouping and Intervention Methods

RAW264.7 macrophages were randomly divided into a blank group (BG), a model group (MG), and low‐dose (LDG), medium‐dose (MDG), and high‐dose (HDG) Sini Decoction‐medicated serum groups, with three independent biological replicates per group. The blank group was routinely cultured without any stimulation or drug treatment, while the model group was treated with 1 μg/mL lipopolysaccharide (LPS) for 24 h to establish an inflammatory injury model in macrophages.

Preparation and quality control of Sini Decoction‐medicated serum: according to the classical formula, the crude medicinal materials of Sini Decoction were weighed, decocted twice with water, and the combined filtrates were concentrated under reduced pressure and lyophilized to obtain the lyophilized powder of Sini Decoction. Healthy rats were continuously administered the decoction by gavage, while blank rats received an equal volume of normal saline in parallel. After the final administration, blood was collected from the abdominal aorta, and serum was separated, inactivated in a 56°C water bath for 30 min, filtered through a 0.22 μM membrane for sterilization, aliquoted, and stored at −80°C for later use. Based on the cytotoxicity results from preliminary experiments, the medicated serum was serially diluted to determine three intervention concentrations (low, medium, and high), and blank rat serum was diluted in parallel as a control.

After 24 h of LPS stimulation, cells in the LDG, MDG, and HDG groups were incubated with corresponding concentrations of Sini Decoction‐medicated serum for an additional 24 h, while the blank and model groups received an equal volume of blank rat serum. At the end of all interventions, cell culture supernatants and total cell lysates were collected for subsequent detection of inflammatory cytokines, macrophage phenotype markers, and mitophagy‐related proteins.

### 2.4 Real‐Time PCR Method

Real‐time PCR was adopted to detect IL‐6, TNF‐α, IL‐10, CD16/32, COX2, Arg1, CD206 and NCOR1. Total RNA isolation was performed using the TRIzol method. Following the manufacturer's protocol, with subsequent quantification of RNA was quantified, and purity was assessed. The RNA was first converted into cDNA through a reverse transcription process, which is essential for downstream applications. This process involves the use of a reverse transcriptase enzyme, which synthesizes a complementary DNA strand from the RNA template. Subsequently, gene amplification was carried out using SYBR Green dye in a quantitative PCR setup, with GAPDH serving as the reference gene. The amplification program included pre‐denaturation, cycling amplification, and concluding with melt curve analysis to validate amplification specificity. Finally, the relative gene expression was quantified using the 2^−ΔΔCt^ approach, and each experiment was meticulously conducted in triplicate, ensuring statistical robustness. This approach not only provided a sufficient sample size for statistical analysis but also helped to minimize the impact of random variations and potential outliers.

### 2.5 Western Blotting (WB) Method

WB was adopted for detecting mitophagy‐related proteins (LC3, Parkin, P62, NCOR1, and FUNDC1). Cellular protein extraction was systematically performed using ice‐cold RIPA lysis buffer (supplemented with protease/phosphatase inhibitors) through a standardized protocol: Following 30‐min lysis on ice, centrifugation of the lysates was carried out at 12,000 rpm for 10 min to pellet cellular debris, with subsequent collection of clarified supernatants as samples for downstream analyses. The protein density was then subjected to measurement using the BCA method. After adjusting the protein concentration to be consistent across all groups, the sample was combined with loading buffer and denatured at 95°C for 5 min. The processed sample was subjected to separation by SDS‐PAGE gel electrophoresis and transfer to a PVDF membrane, and the membrane was incubated with 5% skim milk at 25°C for 1 h. Primary antibodies (Ab) (LC3, Parkin, P62, FUNDC1, and reference GAPDH Ab) were applied, incubated overnight at 4°C, ensuring thorough binding of the Ab to their targets. Primary antibodies against LC3, Parkin, P62, FUNDC1, NCOR1, and the internal reference GAPDH were purchased from Cell Signaling Technology (CST), with catalog numbers #4108, #4211, #5114, #49240, #5948, and #5174, respectively. All primary antibodies were used at a 1:1000 dilution. The horseradish peroxidase‐conjugated secondary antibody (catalog #7074, CST) was applied at a 1:5000 dilution. The membranes were incubated with primary antibodies overnight at 4°C. Following the incubation period, three rinses were carried out with TBST, enhancing the specificity of the subsequent detection process, followed by the addition of the HRP‐conjugated secondary Ab and incubation at 25°C for 1 h. After washing the membrane three times, the bands were visualized using ECL chemiluminescent reagent and recorded by a gel imaging system. Finally, the band intensity was analyzed using ImageJ software, and the gray value ratios of the target to GAPDH were calculated for quantitative contrast. The experiment was carried out in triplicate, ensuring statistical robustness and reproducibility of the findings.

### 2.6 MF Detection

RAW 264.7 macrophages were seeded in Seahorse XF96 cell culture plates at a density of 1 × 10^4^ cells per well and cultured under routine conditions. Upon reaching 70%–80% confluence, preliminary experiments were performed to optimize seeding density, culture medium conditions, and drug injection concentrations. Before the assay, the original complete medium was removed and replaced with pre‐warmed Seahorse XF base medium supplemented with 1 mM sodium pyruvate, 2 mM glutamine, and 10 mM glucose. The plates were then incubated at 37°C in a CO_2_‐free incubator for 1 h to allow equilibration and stabilization of the extracellular microenvironment. The standard mitochondrial stress test program was applied, during which the instrument automatically injected three mitochondrial modulators in sequence: oligomycin (ATP synthase inhibitor), FCCP (mitochondrial uncoupler), and rotenone combined with antimycin A (complex I/III inhibitors). Real‐time dynamic values of OCR and ECAR were continuously recorded for each group throughout the entire procedure.

After the assay, cells in each well were lysed, and total protein content was determined using the BCA method. All raw OCR and ECAR values were normalized to the corresponding total protein amount to correct for well‐to‐well variations in cell seeding density. The entire Seahorse mitochondrial function assay was performed with three independent biological replicates to ensure reproducibility of the results.

### 2.7 CV Detection

CV of RAW 264.7 cells was subjected to assessment using the MTS assay. The cells were seeded in the plate with 96 wells and treated according to experimental grouping. After incubation for the designated time (usually 24 h), the MTS reagent was applied at a certain dilution ratio, incubation for an additional 2–4 h. The MTS reagent is metabolized by cells to produce a soluble product, forming a purple solution. Absorbance measurements (OD) were acquired at 490 nm. The experiment was carried out in triplicate to minimize experimental variability.

### 2.8 Statistical Processing

SPSS 24.0 statistical software was employed. Normally distributed measurement data were represented as mean ± SD ($\bar{x}$ ± s), and categorical data were represented as *n* and %. The Mann‐Whitney test was adopted for the distribution of measurement data that did not follow a normal distribution, while one‐way analysis of variance was adopted for normally distributed measurement data. Pairwise comparisons between groups were corrected by the LSD method for multiple comparisons. χ^2^ test was adopted for the contrast of categorical data. The distinction observed was found to be statistically meaningful, as indicated by a *P*‐value less than 0.05. Three independent replicates were set for all experiments to ensure the reliability of statistical results.

## 3 Results

### 3.1 Changes in Pro‐Inflammatory Cytokine mRNA Expression Across Groups

As shown in Figure 1, compared with the BG, the mRNA expression levels of IL‐6 and TNF‐α in the MG were significantly increased (*p* = 0.002 and *p* = 0.003, respectively). Compared with the MG, the expression of IL‐6 and TNF‐α progressively decreased with increasing drug doses in the LDG, MDG, and HDG groups: LDG (IL‐6, *p* = 0.041; TNF‐α, *p* = 0.038), MDG (IL‐6, *p* = 0.009; TNF‐α, *p* = 0.014), and HDG (IL‐6, *p* = 0.001; TNF‐α, *p* = 0.002). No statistically significant differences were observed between the HDG and BG groups for any of the parameters (all *p* > 0.05).

**Figure 1:**
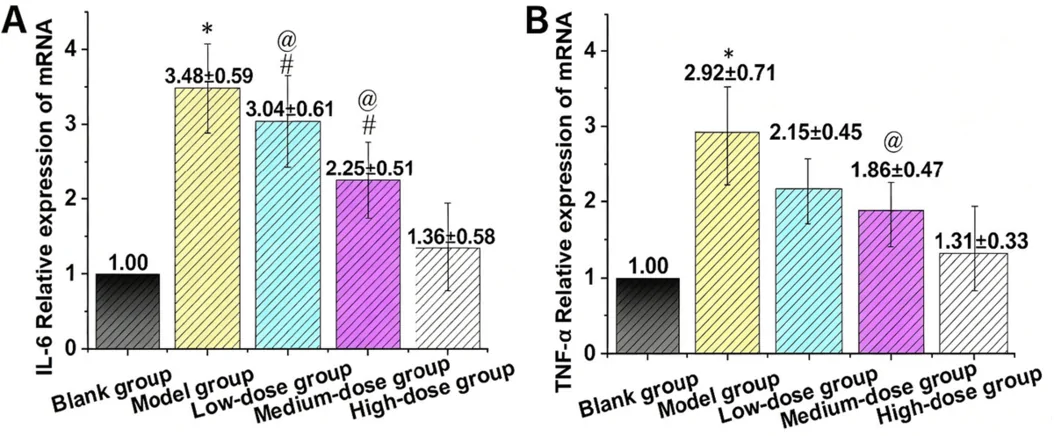
Contrast of inflammatory cytokine expression. (A‐B represent IL‐6 and TNF‐α, respectively). *compared with BG, ^#^compared with MG, @ compared with HDG, *p* < 0.05, BG, blank group; HDG, high‐dose group; HDG, high‐dose group; IL‐6, interleukin‐6; LDG, low‐dose group; MDG, medium‐dose group; MG, model group; TNF‐α, tumor necrosis factor‐α.

### 3.2 Changes in Anti‐Inflammatory Cytokine IL‐10 mRNA Expression Across Groups

As shown in Figure 2, compared with the BG, the mRNA expression level of IL‐10 in the MG was significantly decreased (*p* = 0.001). Compared with the MG, IL‐10 expression in the LDG, MDG, and HDG groups increased progressively with increasing drug doses in a dose‐dependent manner: LDG (*p* = 0.036), MDG (*p* = 0.012), and HDG (*p* = 0.002). No statistically significant difference was observed between the HDG and BG groups (*p* > 0.05).

**Figure 2:**
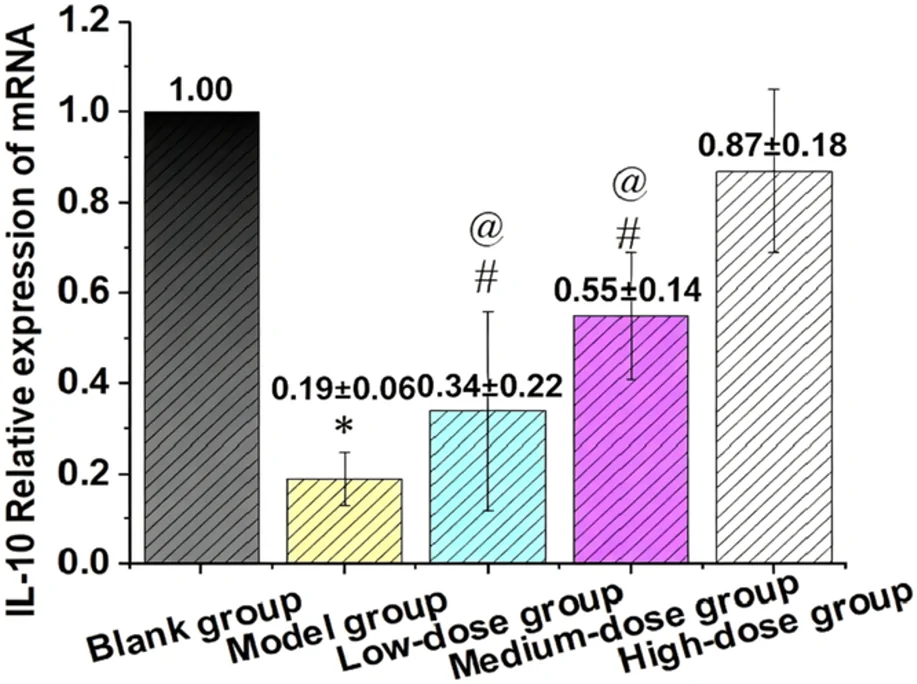
Contrast of anti‐inflammatory cytokine expression. *compared with BG, ^#^compared with MG, @ compared with HDG, *p* < 0.05; BG, blank group; HDG, high‐dose group; IL‐10, interleukin‐10; interleukin‐10; LDG, low‐dose group; MG, model group; MDG, medium‐dose group.

### 3.3 Changes in M1 Macrophage Marker mRNA Expression Across Groups

Compared with the BG (Figure 3), the mRNA expression levels of CD16/32 (*p* = 0.018) and COX2 (*p* < 0.001) in the MG were significantly increased. Compared with the MG, with increasing drug concentrations, the expression of CD16/32 and COX2 in the LDG, MDG, and HDG groups gradually decreased: LDG (CD16/32, *p* = 0.043; COX2, *p* = 0.035), MDG (CD16/32, *p* = 0.016; COX2, *p* = 0.003), and HDG (CD16/32, *p* = 0.008; COX2, *p* < 0.001). No statistically significant differences were observed between the HDG and BG groups for any of the parameters (all *p* > 0.05).

**Figure 3:**
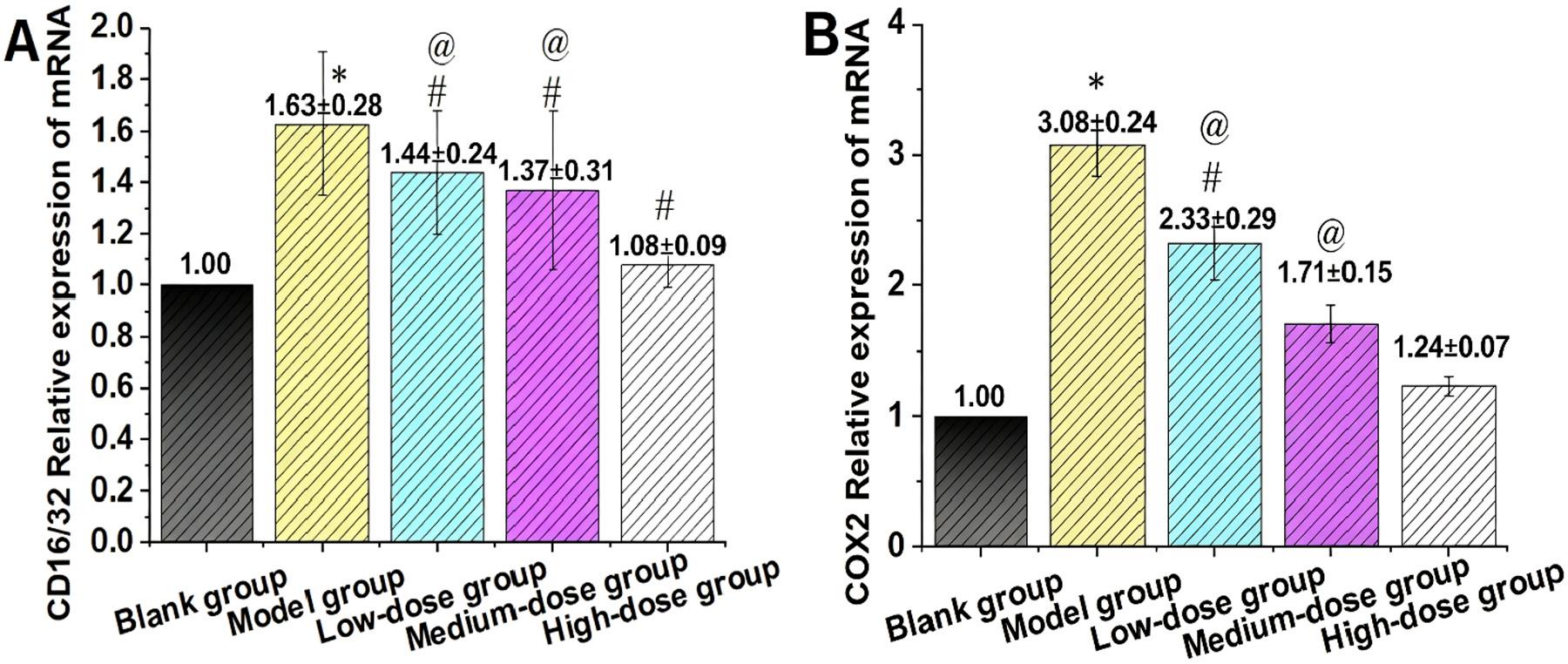
Contrast of M1 macrophage markers. (A‐B represent CD16/32 and COX2, respectively). *compared with BG, ^#^compared with MG, @ compared with HDG, *p* < 0.05; BG, blank group; COX2, cyclooxygenase 2; HDG, high‐dose group; LDG, low‐dose group; MG, model group; MDG, medium‐dose group.

### 3.4 Changes in M2 Macrophage Marker mRNA Expression Across Groups

Compared with the BG (Figure 4), the mRNA expression levels of Arg1 (*p* = 0.001) and CD206 (*p* = 0.002) in the MG were significantly decreased. Compared with the MG, with increasing drug concentrations, the expression of Arg1 and CD206 in the LDG, MDG, and HDG groups gradually increased: LDG (Arg1, *p* = 0.034; CD206, *p* = 0.044); MDG (Arg1, *p* = 0.012; CD206, *p* = 0.016); HDG (Arg1, *p* = 0.002; CD206, *p* = 0.001). No statistically significant differences were observed between the HDG and BG groups for any of the parameters (all *p* > 0.05).

**Figure 4:**
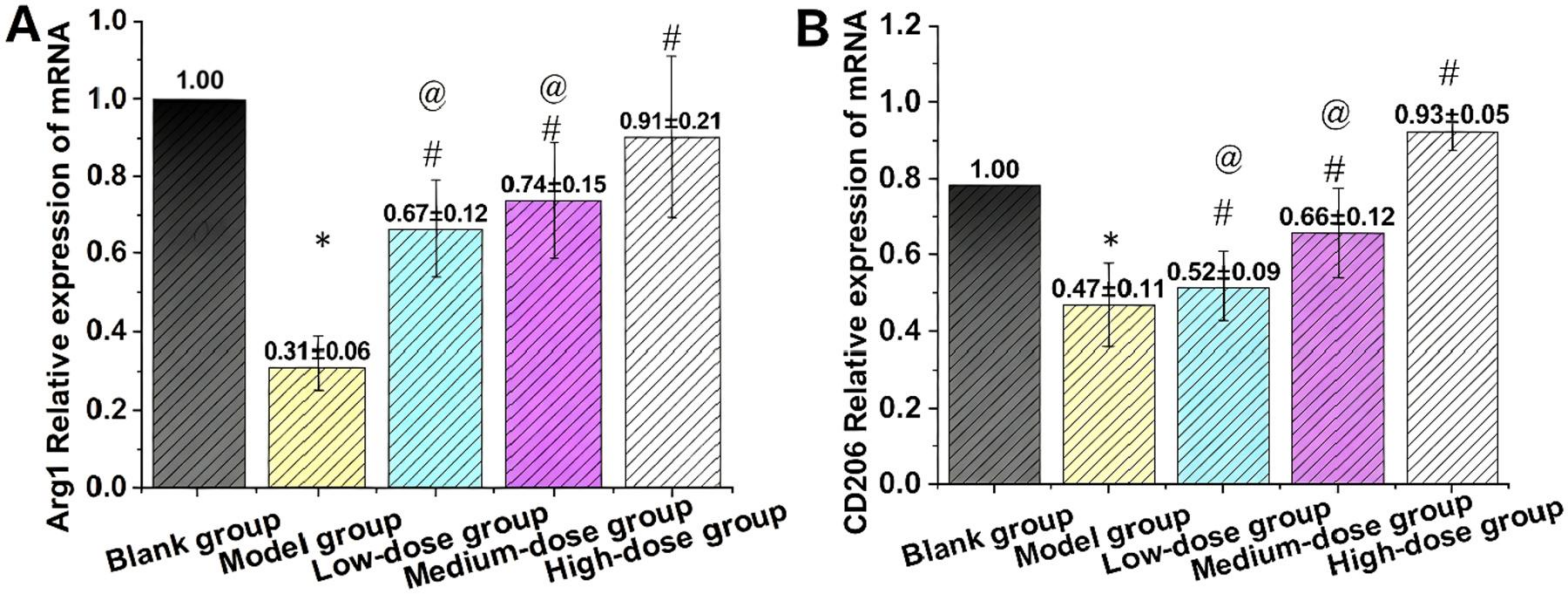
Contrast of M2 macrophage markers. (A‐B represent Arg1 and CD206, respectively). *compared with BG, ^#^compared with MG, @ compared with HDG, *p* < 0.05; Arg1, arginase‐1; BG, blank group; HDG, high‐dose group; LDG, low‐dose group; MG, model group; MDG, medium‐dose group.

### 3.5 Changes in Mitophagy‐Associated Protein Expression Across Groups

Compared with the BG (Figures 5 and 6), the protein expression levels of LC3 (*p* = 0.002), Parkin (*p* < 0.001), and FUNDC1 (*p* = 0.003) in the MG were significantly increased, while P62 expression (*p* = 0.004) was significantly decreased. Compared with the MG, with increasing drug concentrations, the expression of LC3, Parkin, and FUNDC1 in the LDG, MDG, and HDG groups gradually decreased, whereas P62 expression gradually increased: LDG (LC3, *p* = 0.041; Parkin, *p* = 0.033; FUNDC1, *p* = 0.039; P62, *p* = 0.042); MDG (LC3, *p* = 0.011; Parkin, *p* = 0.008; FUNDC1, *p* = 0.015; P62, *p* = 0.013); HDG (LC3, *p* = 0.003; Parkin, *p* = 0.001; FUNDC1, *p* = 0.004; P62, *p* = 0.003). No statistically significant differences were observed between the HDG and BG groups for any of the detected proteins (all *p* > 0.05).

**Figure 5:**
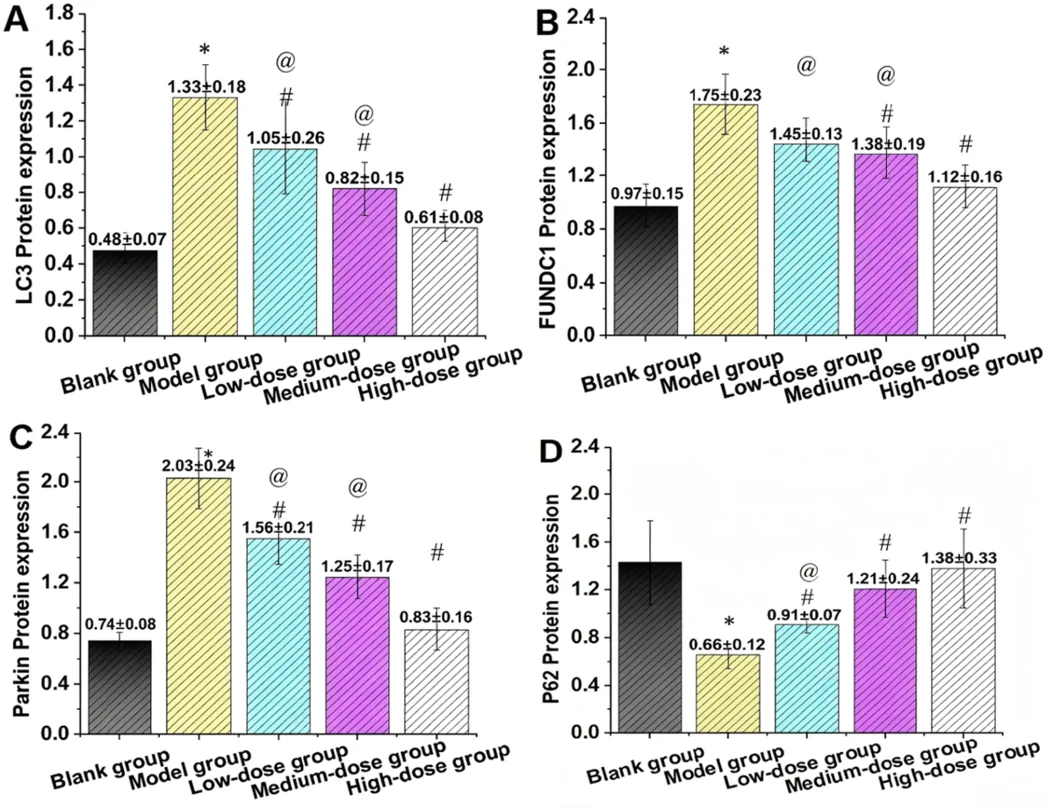
Contrast of mitophagy‐related protein expression. (A‐D represent LC3, Parkin, FUNDC1, and P62, respectively). *compared with BG, ^#^compared with MG, @ compared with HDG, *p* < 0.05; BG, blank group; FUNDC1, FUN14 domain‐containing 1; HDG, high‐dose group; LC3, microtubule‐associated protein light chain 3; LDG, low‐dose group; MDG, medium‐dose group; MG, model group; P62, sequestosome‐1.

**Figure 6:**
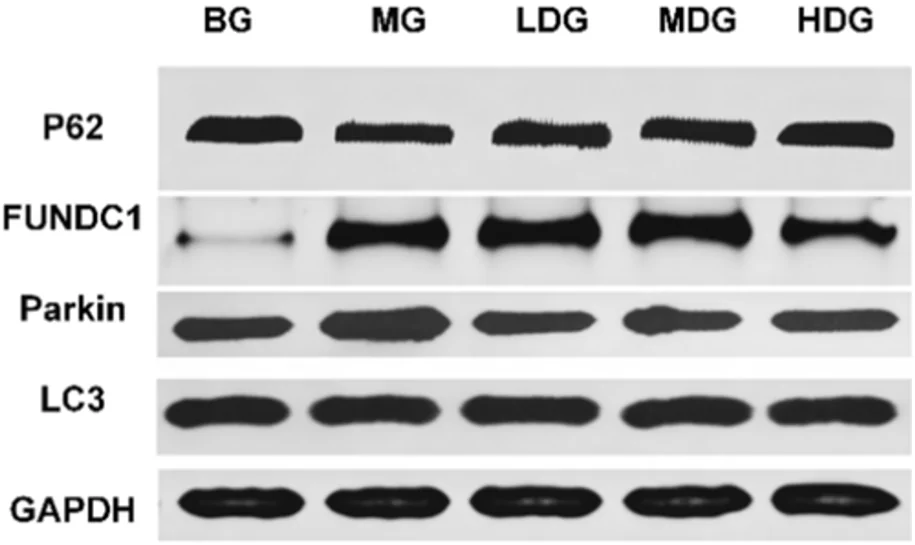
Western blot bands of p62, fundc1, parkin and LC3 proteins of cells in each group. BG, blank group; FUNDC1, FUN14 domain‐containing 1; HDG, high‐dose group; LC3, microtubule‐associated protein light chain 3; LDG, low‐dose group; MG, model group; MDG, medium‐dose group; P62, sequestosome‐1.

### 3.6 Changes in Mitochondrial Function Indicators OCR and ECAR Across Groups

Compared with the BG (Figure 7), the OCR level in the MG was significantly decreased (*p* < 0.001), while the ECAR level was significantly increased (*p* < 0.001). Compared with the MG, with increasing drug concentrations, the OCR level in the LDG, MDG, and HDG groups gradually increased, whereas the ECAR level gradually decreased: LDG (OCR, *p* = 0.037; ECAR, *p* = 0.031); MDG (OCR, *p* = 0.012; ECAR, *p* = 0.008); HDG (OCR, *p* = 0.002; ECAR, *p* = 0.003). No statistically significant differences in OCR or ECAR levels were observed between the HDG and BG groups (all *p* > 0.05).

**Figure 7:**
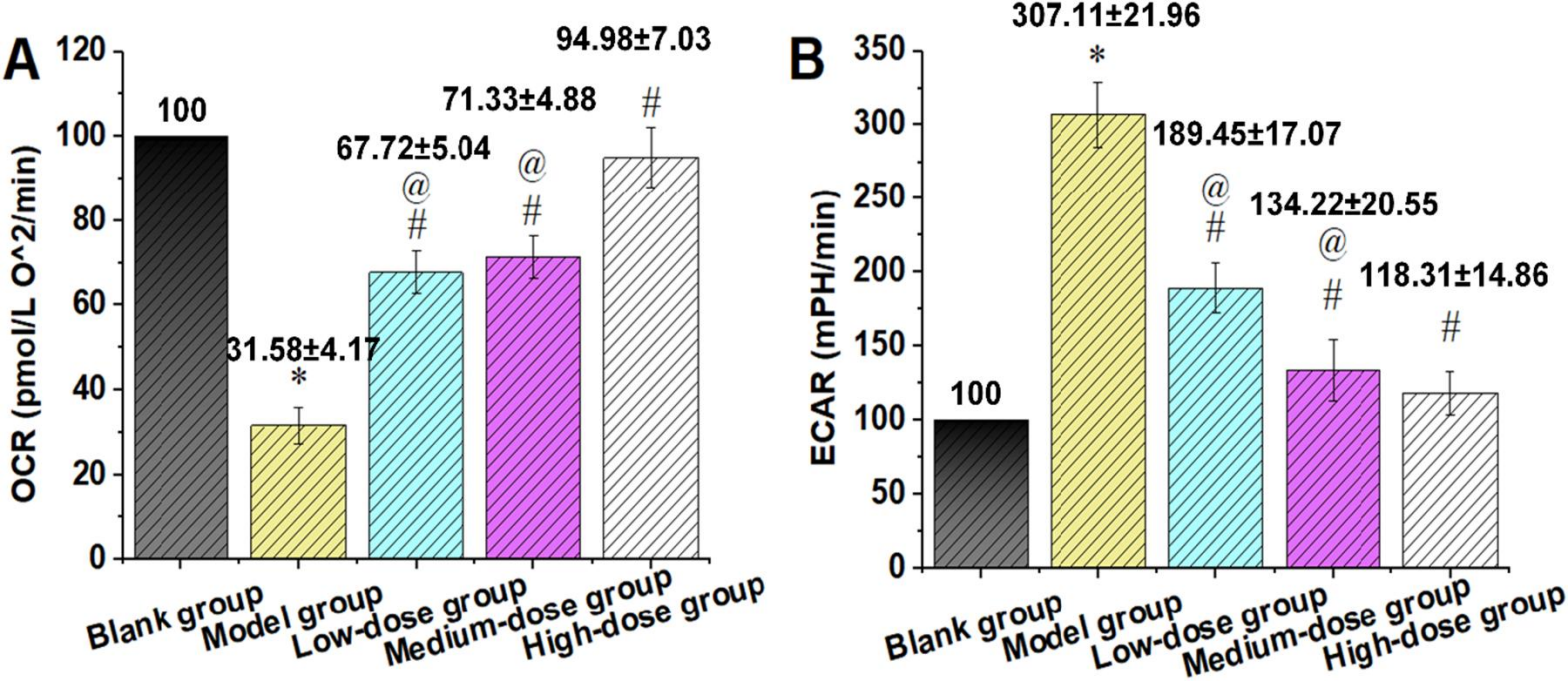
Contrast of MF indicators. (A: OCR; B: ECAR). * compared with BG, # compared with MG, @ compared with HDG, *p* < 0.05; BG, blank group; ECAR, extracellular acidification rate; HDG, high‐dose group; LDG, low‐dose group; MG, model group; MDG, medium‐dose group; OCR, oxygen consumption rate.

### 3.7 Changes in Cell Viability Among all Experimental Groups

As shown in Figure 8, compared with the BG group, cell viability in the MG group was significantly reduced (*p* < 0.001). Compared with the MG group, cell viability in the LDG, MDG, and HDG groups increased progressively with increasing drug concentrations: LDG (*p* = 0.039), MDG (*p* = 0.011), and HDG (*p* = 0.002). No statistically significant difference in cell viability was observed between the HDG and BG groups (*p* > 0.05).

**Figure 8:**
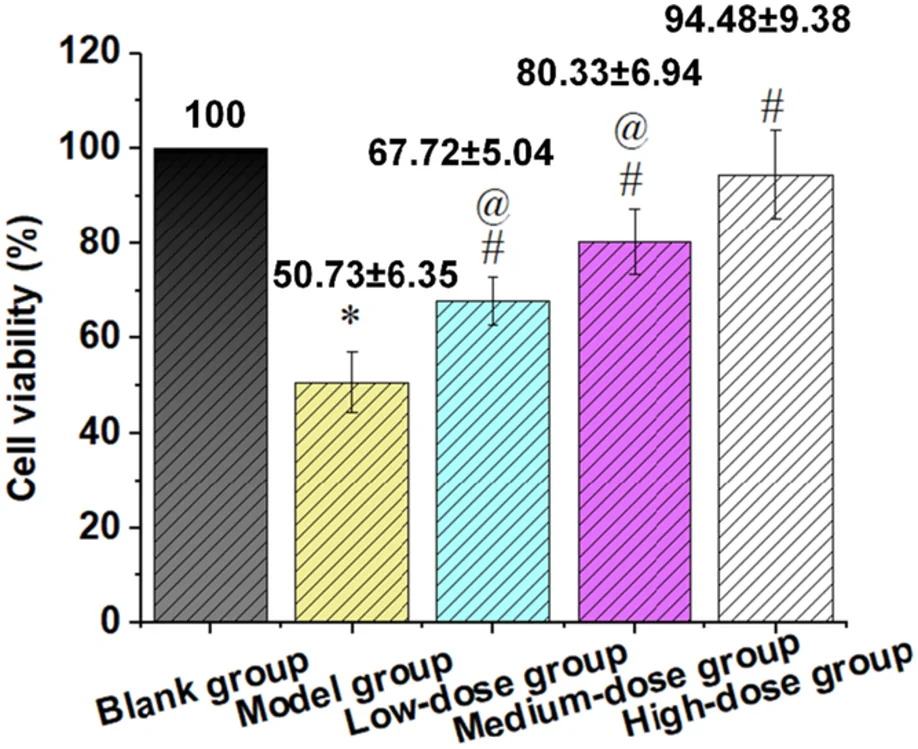
Contrast of CV. *compared with BG, ^#^compared with MG, @ compared with HDG, *p* < 0.05; BG, blank group; CV, cell viability; HDG, high‐dose group; LDG, low‐dose group; MG, model group; MDG, medium‐dose group.

### 3.8 Relative Expression of NCOR1 mRNA and Protein in Each Group

As shown in Table 1, compared with the BG group, the NCOR1 mRNA expression level in the MG group was significantly decreased (*p* = 0.002). Compared with the MG group, the NCOR1 mRNA expression in the LDG, MDG, and HDG groups gradually recovered with increasing drug concentrations, exhibiting a dose‐dependent trend: LDG (*p* = 0.036), MDG (*p* = 0.010), and HDG (*p* = 0.002). No statistically significant difference was observed between the HDG and BG groups (*p* > 0.05).

**Table 1:** Relative expression levels of ncor1 mRNA in macrophages of each group.

Grouping	NCOR1 mRNA relative expression
BG	1.00 ± 0.08
MG	0.36 ± 0.05*
LDG	0.54 ± 0.06^#^
MDG	0.78 ± 0.07^#^
HDG	0.95 ± 0.09^#^

*Note:* *represents the comparison with the blank group, *p* < 0.05; ^#^represents the comparison with the model group, *p* < 0.05.

Abbreviations: BG, blank group; HDG, high‐dose group; LDG, low‐dose group; MG, model group; MDG, medium‐dose group; NCOR1, nuclear receptor corepressor 1.

The results of NCOR1 protein expression in each group are presented in Figure 9 and Table 2. The protein expression trend was consistent with the mRNA findings. Relative to the BG group, NCOR1 protein expression in MG cells was significantly downregulated (*p* = 0.003). After intervention with different concentrations of Sini Decoction‐containing serum, NCOR1 protein expression in the LDG, MDG, and HDG groups increased progressively compared with the MG group, showing a clear dose‐dependent effect: LDG (*p* = 0.041), MDG (*p* = 0.008), and HDG (*p* = 0.002). No significant difference in NCOR1 protein expression was found between the HDG and BG groups (*p* > 0.05).

**Figure 9:**
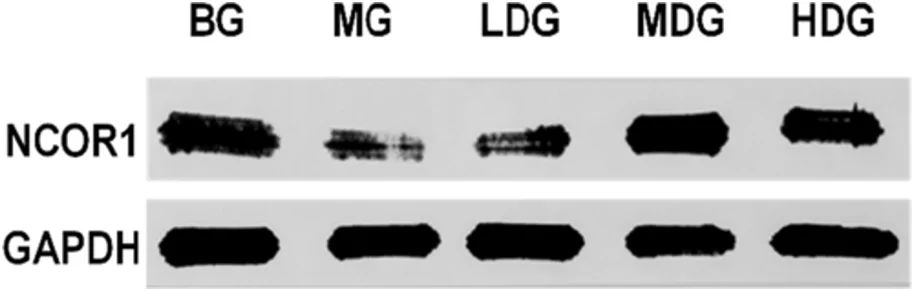
Western blot bands of ncor1 protein in each group. ncor1 protein WB original electrophoresis band, GAPDH as internal reference; From left to right, lanes are BG, MG, LDG, MDG, HDG. BG, blank group; GAPDH, glyceraldehyde‑3‑phosphate dehydrogenase; HDG, high‑dose group; LDG, low‑dose group; MG, model group; MDG, medium‑dose group; NCOR1, nuclear receptor corepressor 1.

**Table 2:** Relative expression levels of ncor1 protein in macrophages of each group.

Grouping	Ncor1/gapdh relative gray value
BG	0.97 ± 0.07
MG	0.31 ± 0.04*
LDG	0.49 ± 0.05^#^
MDG	0.74 ± 0.06^#^
HDG	0.92 ± 0.08^#^

*Note:* *represents the comparison with the blank group, *p* < 0.05; ^#^represents the comparison with the model group, *p* < *0.05*.

Abbreviations: BG, blank group; HDG, high‐dose group; LDG, low‐dose group; MG, model group; MDG, medium‐dose group; NCOR1, nuclear receptor corepressor 1.

## 4 Discussion

AS is a chronic inflammatory disease closely related to the occurrence of cardiovascular diseases. Macrophages play a crucial role in the pathogenesis of AS. The polarization of macrophages, inflammatory responses, and their regulation of MF are key processes in the development of AS. SND, as a traditional Chinese herbal formula, has anti‐inflammatory, antioxidant, and immune‐regulating effects and has been widely studied for its therapeutic potential in various diseases. However, the mechanisms by which SND regulates macrophage polarization and mitophagy have not been fully explored [17]. This article investigated the effects of SND on macrophage polarization and MF in an LPS‐induced macrophage AS model and revealed its possible molecular mechanisms. The inflammatory cytokines are important mediators in the pathogenesis of AS. IL‐6 is involved not only in the activation of macrophages but is also closely related to lipid accumulation and plaque formation. TNF‐α further exacerbates the progression of AS by promoting the secretion of adhesion molecules by vascular endothelial cells [18]. In this article, SND inhibited the inflammatory response of macrophages, suggesting that it may exert anti‐atherosclerotic effects by reducing systemic inflammatory responses. SND increased the levels of IL‐10 in RAW 264.7 cells after LPS stimulation, especially in the HDG. IL‐10, by activating the STAT3 signaling pathway, inhibits the polarization of M1 macrophages and promotes the transformation of M2 macrophages, thereby exerting anti‐inflammatory effects in the immune response of AS. The regulatory effect of SND on IL‐10 further suggests that it promotes the transformation of macrophages to an anti‐inflammatory phenotype, which helps to reduce the inflammatory response in AS.

M1 macrophages are considered the main pro‐inflammatory cell type in AS, exacerbating local inflammatory responses through the secretion of large amounts of inflammatory cytokines [19]. Studies have shown that the role of COX2 in AS is to promote inflammatory responses. By inhibiting the activation of M1 macrophages, SND may alleviate atherosclerosis‐related inflammatory damage at the cellular level and confer potential benefits on pathological changes associated with plaque formation. In addition, SND may mitigate AS‐associated macrophage‐mediated inflammatory lesions through promoting M2 macrophage polarization, thereby exerting protective effects at the cellular level and contributing to the reduction of vascular inflammatory injury [20].

Mitophagy is a key mechanism. In this article, SND markedly regulated the expression of LC3, Parkin, P62, and FUNDC1. LC3 and Parkin are key molecules in mitophagy; the upregulation of LC3 indicates raised autophagic activity, while the upregulation of Parkin suggests enhanced clearance of damaged mitochondria. P62, a bridging protein involved in intracellular waste clearance during mitophagy, is typically associated with raised autophagic activity when upregulated. FUNDC1, an essential receptor in the autophagic process, is involved in selective mitophagy [21]. Sini Decoction can alter the expression levels of mitophagy‐related proteins (LC3, Parkin, FUNDC1, and P62) in LPS‐stimulated macrophages and restore mitochondrial function. However, this study only examined static protein expression and did not perform mitophagic flux monitoring; upregulation of P62 cannot be directly interpreted as enhanced mitophagy, as it may also suggest a blockade of the autophagic degradation pathway for damaged mitochondria. MF is central to cellular energy metabolism and is vital for the activation and polarization of macrophages. In this article, SND markedly improved the OCR and ECAR of macrophages after LPS stimulation. The increase in OCR indicates improved aerobic metabolism, while the decrease in ECAR suggests reduced glycolytic activity, indicating that SND helps restore normal energy metabolism in macrophages [22]. Studies have shown that metabolic reprogramming of macrophages is closely related to their function, and SND may improve cellular physiological function by regulating the metabolic state of macrophages. In this article, SND markedly enhanced the viability of macrophages after LPS stimulation. MTS assay results showed that CV gradually recovered with increasing doses of SND, and there was no significant distinction in CV between the HDG and the BG. The recovery of CV not only reflects the reparative effect of SND on macrophage damage but also further confirms its protective effect on cellular function. qPCR and Western blot results collectively confirmed that LPS significantly inhibited the gene transcription and protein expression of NCOR1 in macrophages, and that Sini Decoction‐containing serum reversed this inhibitory effect in a dose‐dependent manner. Combined with the altered expression of mitophagy‐related proteins, we speculate that the regulatory effects of Sini Decoction on macrophage polarization and inflammation may be related to the NCOR1 pathway. However, due to the lack of mitophagic flux measurement and NCOR1 gene intervention experiments, it is not yet possible to confirm that Sini Decoction exerts anti‐inflammatory and plaque‐stabilizing effects by upregulating NCOR1 to regulate mitophagy. Moreover, this study only conducted in vitro cellular experiments, which cannot directly infer its actual effects on stabilizing atherosclerotic plaques in vivo.

This study relied solely on RAW264.7 macrophages for in vitro investigation, without establishing an animal model of lower extremity atherosclerosis. A single‐cell culture environment cannot fully recapitulate the complex physiological microenvironment of human blood vessels, which contains multiple cell types and inflammatory cytokines. Moreover, the lack of clinical specimens from patients with lower extremity atherosclerosis makes it difficult to evaluate the actual effects of Sini Decoction in humans, and to some extent weakens the translational relevance of the experimental conclusions to clinical practice. In this experiment, Sini Decoction‐containing serum was used as the intervention medium, without further isolation of the active monomers from the formula; thus, the core pharmacologically active substances mediating macrophage polarization and mitophagy regulation were not identified. Additionally, only the NCOR1 mRNA expression level was measured, and no gene intervention validation was performed, so the complete upstream and downstream regulatory pathways of NCOR1 have not been confirmed. The gradient design of the experiment has certain limitations: the concentration groups of drug‐containing serum were few and the concentration intervals were relatively wide, making it difficult to precisely identify the optimal intervention dose. The detection techniques only covered PCR, Western blot, mitochondrial function, and cell viability assays, lacking reverse validation approaches such as immunofluorescence and gene rescue experiments, leaving gaps in the overall molecular evidence chain. About mitophagy detection, this study only measured the static protein expression levels of LC3, Parkin, FUNDC1, and p62, without performing mitophagic flux assays. Consequently, it is impossible to distinguish whether the changes in protein expression reflect upregulated initiation of mitophagy or blockage of the downstream degradation pathway. The increased p62 expression in the Sini Decoction intervention group may be interpreted in two completely different ways, which limits the reliability of the mitophagy‐related inferences. To address these objective limitations, subsequent studies will establish an in vivo atherosclerotic model in ApoE^−^/^−^ mice to corroborate the in vitro findings at the animal level. We will isolate and purify the characteristic active components of Sini Decoction and supplement with NCOR1 gene intervention and rescue experiments. We will also employ mt‐Keima fluorescent probes and bafilomycin A1 blockade assays to detect mitophagic flux and clarify the dynamic changes in mitophagy. Simultaneously, we will collect clinical vascular specimens to establish a combined in vitro and in vivo validation system, thereby comprehensively strengthening the existing experimental evidence of this study.

## 5 Conclusion

In this study, an LPS‐induced RAW264.7 macrophage inflammatory model was established to investigate the regulatory effects of Sini Decoction‐containing serum on macrophage polarization, mitophagy, and mitochondrial metabolism at the cellular level, and to preliminarily observe its impact on NCOR1 mRNA and protein expression. The in vitro results demonstrated that Sini Decoction‐containing serum dose‐dependently inhibited the pro‐inflammatory response of macrophages, promoted polarization toward the M2 anti‐inflammatory phenotype, ameliorated abnormal mitophagy and mitochondrial function, and simultaneously upregulated NCOR1 expression. These cytological findings suggest that Sini Decoction possesses potential effects in intervening in atherosclerosis‐related inflammation, and this regulatory effect may be associated with NCOR1. The above cytological evidence reveals the potential value of SND in intervening in atherosclerosis‐related inflammatory lesions; however, further in vivo animal models with intact plaque structures and clinical specimens are still required to verify whether SND can exert plaque‐stabilizing effects within blood vessels. This study provides in vitro molecular experimental evidence for subsequent in‐depth research on the anti‐atherosclerotic effects of SND.

## Author Contributions

Zhe Liu, Shuhui Ge, Jianfei Yang designed the study; all authors conducted the study; YingZhou collected and analyzed the data. Li Jin, Yudong Fang participated in drafting the manuscript, and all authors contributed to critical revision of the manuscript for important intellectual content. All authors gave final approval of the version to be published. All authors participated fully in the work, took public responsibility for appropriate portions of the content, and agreed to be accountable for all aspects of the work in ensuring that questions related to the accuracy or completeness of any part of the work were appropriately investigated and resolved.

## Ethics Statement

The animal experimental protocol used in this study for the preparation of Sini Decoction‐containing serum was approved by the Animal Ethics Committee of Shanghai TCM‐Integrated Hospital, Shanghai University of Traditional Chinese Medicine (Ethics Approval No. GENINK‐20250034). All procedures involving animal housing, drug administration, and blood collection were strictly performed in accordance with the guidelines of the *Guidelines for the Welfare and Ethics of Laboratory Animals*. This in vitro cell experiment did not require clinical specimens from patients and did not involve any aspects related to human informed consent.

## Conflicts of Interest

The authors declare no conflicts of interest.

## Data Availability

The datasets used and/or analysed during the current study were available from the First author on reasonable request.
